# The Advance on Frontotemporal Dementia (FTD)'s Neuropathology and Molecular Genetics

**DOI:** 10.1155/2022/5003902

**Published:** 2022-10-13

**Authors:** Jindong Wang, Bailing Wang, Tiantian Zhou

**Affiliations:** Department of Geriatric Psychiatry, Qingdao Mental Health Center, Shandong 266034, China

## Abstract

The morbidity of frontotemporal dementia (FTD), one of the most prevalent dementias praccox, is second to Alzheimer disease (AD). It is different with AD that FTD has a rapider course and a higher mortality. FTD has not yet been fully understood in terms of etiology or pathogenesis, but genetic factors are believed to be involved. In this paper, we were committed to providing a comprehensive overview to FTD in aspects of the neuropathology features and the relevant molecular genetics advances, so that there would be insights to those researchers in search of novel approaches in FTD diagnosis and treatment.

## 1. Introduction

There are approximately 20% of the presenile dementia patients diagnosed with FTD, and those people represent a series of clinical neurologic syndromes featured with early-onset relatively retained memory, progressive behavioral abnormalities, personality changes, and language disorders [1, 2]. Studies on FTD revealed a heterogeneity in neuromolecular pathology; besides, different subtypes of FTD syndromes possibly vary in their etiology and pathogenesis [3]. Nevertheless, there were still great progress made in the studies on FTD neuropathology and pathogenesis in recent years. FTD is the second most common neurodegenerative dementia in younger patients (<65 years old), whose onset has conspicuous impact on the patients' life expectancy [4, 5]. It was reported that those patients' survival time after onset was only about 3 to 14 years. In contrast, FTD patients are estimated to bear a two- to three-fold greater economic burden than AD patients [6, 7].

Clinically speaking, syndromes of FTD are categorized into behavioral variant frontotemporal dementia (bvFTD), the behavioral type; primary progressive aphasia (PPA), the linguistic variant type; and FTD amyotrophic lateral sclerosis (FTD-ALS/atypical Parkinson's disease), the sportive manifestation type [8, 9]. Neuroanatomically speaking, FTD is in a characteristic relation to the functional disorders and to the neuron loss of both the frontal and temporal lobes, even broad to cortex, subcortex, cerebellum, and brain stem. Genetically, approximately one-third of FTDs are familial, with autosomal dominant mutations in three genes accounting for the majority of inheritance: progranulin (GRN), chromosome 9 open reading frame 72 (C9orf72), and microtubule- associated protein tau (MAPT). Pathologically speaking, cellular inclusions of abnormal forms of tau, TDP-43, or FET (fused in sarcoma (FUS), Ewing's sarcoma, TATA-binding protein-associated factor 15) proteins are found in majority of FTD cases, which implies the correlation of mutant protein with FTD.

## 2. The Research Development of FTD Neuropathological Feature

Brain-tissue pathological manifestations of most FTD patients are localized atrophy of bilateral frontal and anterior temporal lobes, expansion of cerebral ventricle, and possible involvements in both gray matter and white matter. There are also a certain of impairments in hippocampal CA1 region, basal ganglia, amygdala, substantia nigra, and nucleus of brain. Other than that, neuroloss, microvacuolation, gliosis, and spongiform degeneration would be observed under light microscope [10, 11]. Deposition of tau is normal in neurons and glia, but there may not be pathological changes such as senile plaques, neurofibrillary tangles (NFTs), or Lewy bodies.

It was reported that under the microscope, there were about 50% of FTD patients showing transactive response DNA-Bindin protein 43 (TDP-43) aggregation (FTD-TDP), and 45% of FTD patients showing MAPT, namely, FTD-Tau. In addition, FTD featured with aggregation of RNA-binding protein fused in sarcoma (FUS), also known as FTD-FUS, occurred in <5% of cases. FTD-TDP, FTD-Tau, and FTD-FUS are also subdivided into different subtypes on the basis of immunohistochemical characteristics [12, 13].

### 2.1. FTD-TDP

TDP-43 is a widely expressed protein, and it is also a highly evolutionarily conserved member of TDP family encoded by TARDBP gene on chromosome 1, the distribution of which is mainly inside the nucleus. TDP-43 is consisted of 414 amino acid residues with a relative molecular weight of 43 000. TDP-43 protein has two RNA recognition motifs and a C-terminal glycine rich region. Recent studies have found that TDP-43 is regulated by nuclear localisation signal (NLS) and nuclear export signal (NES) motifs; apart from its neuronal regulation activity, it also constantly shuttles between the cytoplasm and the nucleus [14, 15]. Transcription and splicing are also involved in the courses of neurons' differentiation and apoptosis to maintain the stability of mRNA, which are the neuronal activity response to modulate neuronal plasticity [16–18].

In terms of the morphology and distribution of pathological inclusion bodies, FTD-TDP could be divided into four pathological subtypes, namely, FTD-TDP1, FTD-TDP 2, FTD-TDP 3, and FTD-TDP4. The main feature of FTD-TDP 1 symptom is that the abundant long neurites in the patients' cerebral cortex superficial layer are without or with only a few neuronal cytoplasmic inclusions (NCIs), neuronal intranuclear inclusions (Nils), and glial cytoplasmic inclusions (GCIs). Different from FTD-TDP 1, there are more NCIs in FTD-TDP 2 patients' cortex superficial and deep layers with normal amount of NCI and GCI precursors; in this context, neurites may be present, but Nils are absent or deficient. FTD-TDP 3 refers to the large number of short neurites and NCIs in the superficial cerebral cortex [19–21]. In addition, there would be a moderate number of crystalline Nils in the pathological cortex, which is more common in patients with a positive family genetic history. The neuropathological characteristics of FTD-TDP4 are the presence of a large number of Nils and dystrophic ncurites (DNs) in the pathological cortex. In this condition, a very small amount of NCIs exist, but there is no pathological inclusion body in the granulosa cell layer of the hippocampus. FTD-TDP also shows different clinical phenotypes, and svPPA and FTD-ALS are closely related to TDP-43 pathology; furthermore, psychiatric symptoms in FTD patients are associated with underlying TDP pathology.

### 2.2. FTD-Tau

Tau misfolding or abnormal aggregation eventually leads to the formation of pathological neuronal inclusion bodies and microtubule instability [22]. FTD-tau is deemed as a diagnostic basis to several neuropathological conditions according to the morphology of the major tau isoforms and the inclusion bodies in such aggregates Thus, FTD-tau includes Pick's disease (PiD) characterized by 3R tau pathology, 4R tau lesions PSP, corticobasal degeneration (CBD), and globular glial tauopathy (GGT). FTD-tau is also classified in lights of different clinical phenotypes. Clinical evidence suggested that almost half of bvFTD cases have underlying FTD-tau pathology, while PiD and few CBD and PSP pathologies are also included. Furthermore, FTD-Tau pathology has also been reported in patients with PPA and corticobasal syndrome (CBS) phenotype [23, 24]. There was also evidence that the clinical syndrome of PSP is highly correlated with PSP tau pathology.

### 2.3. FTD-FUS/UPS

FUS is a multifunctional nuclear DNA/RNA binding protein that has biological effects in transcriptional regulation, RNA transport, and cell growth. Although the amount of FUS inclusion bodies is not normal in FTD, patients with underlying FTD-FUS pathology usually meet the diagnostic criteria for bvFTD. These patients are often clinically characterized by a negative family history and caudate atrophy [25].

The international FTD working group proposed to classify FTD-related neurodegenerative diseases into five different neuropathologies according to the anatomical sites and the pathological changes of clinical signs:

- The first is a neuropathologically abnormal, positive tau inclusion body and obviously insoluble tau tribundle recombination, which is often diagnosed as Pick disease, FTDP-17, or other as yet unidentified familial, sporadic frontotemporal disease
- The second, a neuropathologically abnormal, positive tau inclusion body, significant insoluble tau tetragonal bundle recombination, which is often diagnosed with CBD, PSP, FTDP-17, or other as yet unidentified familial and sporadic frontotemporal disease
- The third, a neuropathologically abnormal, positive tau inclusion bodies, significant insoluble tau recombination of three and four microbundles, is most likely to be diagnosed as neurofibrotangential dementia, FTDP-17, or other as yet unidentified familial and sporadic frontotemporal disease
- The fourth, patients with significant neuropathologic abnormalities with frontal temporal lobe neuronal loss and glial over-growth and with no detectable insoluble tau or tau or ubiquitin positive inclusion bodies detected, are most likely to be diagnosed with DLDH or other with unrecognized familial and sporadic frontotemporal disease
- The fifth, those patients with obvious neuropathologic abnormalities, accompanied by frontal temporal lobe neuron loss and gliosis over-growth, with positive ubiquitin and negative tau inclusion body detected, but with no insoluble tau detected, and accompanied by either motor neuron disease (MND) or without MND, but with MND-type inclusion body detected; then, such patients may be diagnosed with FTD with MND, FTD without MND inclusion bodies, or other with unrecognized familial and sporadic frontotemporal diseases [26, 27].

## 3. Molecular Genetics of FTD

The highly heritable manifestation of FTD is that approximately 10% to 20% of FTD cases are induced by autosomal dominant mutations. Most of these variants are of very poor genotype-phenotypic relevance, but they have unrivalled potential for neuropathological predictability. About one-third of the population is FTD-inherited, and abnormal amplification of C9orf72 is the most common cause, which could be confirmed by repeat primer PCR and Southern blot. Mutations in other common causes (GRN and MAPT) and rare genetic causes, VCP and CHMP2B, are usually identified by second-generation sequencing or exome detection or genome sequencing [28, 29].

### 3.1. C9orf72

Abnormal GGGGCC amplification inside the non-coding region of the C9orf72 gene produces toxic RNA lesions and dipeptide repeat proteins (DPRs), and the number of this amplification is directly related to pathogenicity, with hundreds of such amplification found in most confirmed cases. Most of these carriers have FTD-TDP 1 and FTD-TDP 2 pathology, but the FTD-TDP type 3 is also found. Clinically, FTD C9orf72 carriers might develop either bvFTD or PPA [30, 31].

C9orf72-associated FTD is caused by expansion of the GGGGCC hexanucleotide repeat in the noncoding region of the gene. One of the pathogenic mechanisms is the generation of DPR through repeat-associated non-AUG (RAN) translation: C9orf72 RNA repeats could be translated through RAN translation to generate five DPRs poly(GA), poly(GR), poly(PR), poly(PA), and poly(GP). Many studies have been conducted to measure these DPR levels in cerebrospinal fluid (CSF), but so far only poly (GP) levels was found measurable; moreover, it was found that the level of poly (GP) is increased before and during symptoms, regardless of clinical phenotype or disease stage [32, 33]. Although poly (GP) is not currently available for clinical application, it may be used in practice in a manner similar to that at the level of the granulocyte precursor, making it possible to detect amplification prior to genetic screening. Few relevant proteomic studies have been conducted, so little is known about the interactions between other proteins and the C9orf72 pathway. However, a recent study has compared the CSF proteome of C9orf72-related FTD and C9orf72-related amyotrophic lateral sclerosis (ALS). Moreover, this study also revealed including neurofilament media polypeptide, chitotriosidase, and ubiquitin carboxy-terminal hydrolase isoforms; besides, there are more than 200 proteins differed significantly between the enzyme L1 groups [34–36].

### 3.2. GRN

Over 70 GRN pathogenic mutations have been studied fully to date, and most of them are due to the functional loss caused by abnormal transcription or blocking translation, ultimately leading to insufficient GRN haploid in FTD patients. GRN carriers usually present with FTD-TDP-1 pathology, and most patients have bvFTD clinical phenotype. Occasionally, PPA patients were also found in these familial cases [37, 38].

This precursor protein eventually breaks down into many smaller peptides, i.e., granular proteins 1-7 and para-GRN. These are the key proteins with lysosomal and pro-inflammatory effects to promote TDP-43 accumulation and toxicity. Most of the pathogenic variations in GRN are frameshifting, nonsense, or splicing site mutations that lead to haploid deficiency and ultimately to reduced levels of granuloprotein precursor proteins. Although most studies have been conducted in blood, GRN levels in both blood and CSF are measurable [39, 40].

Other proteins closely related to granuloprotein precursors include prosaposin, sortilin, and secretory leukocyte protease inhibitor (SLPI). Prosaposin is similar to granuloprotein precursors, but it has distinct intracellular and extracellular functions, such as regulation of lysosomal enzymes and neuroprotection of glial cells. Prosaposin is a precursor protein capable of breaking down into four types of saposins that are involved in the breakdown of sphingolipids. The granuloprotein precursor binds to the precursor protein, and both of them are eventually transported to the lysosome. At present, there is no relevant study on prosaposin concentration in FTD. Sortilin, a member of the receptor family in the Vps10p domain, is involved in the endocytosis of granuloprotein precursors into lysosomes, forming key receptors for the function of granuloprotein precursors. The expression level of Sortilin has been measured in biological fluids of aging individuals, and the results showed that Sortilin is strongly positively correlated with the level of granuloprotein precursor in CSF. However, the expression of Sortilin has not been detected in plasma of the same individuals, and there was no relevant study to measure the content of Sortilin in patients with GRN mutations [41, 42]. SLPI is an inhibitor of serine protease and elastase, whose known function is to break down the precursors of granular protein into granular protein.

### 3.3. MAPT

Mutation of MAPT gene (≥40 pathogenic mutations) leads to abnormal tau protein morphology, which not only intensifies tau protein aggregation but also interferes with the aggregation and stability of normal microtubules. These mutations are usually associated with PSP and CBD pathology [43, 44]. Genotype-phenotype correlation showed that MAPT carriers were mainly associated with bvFTD and PPA. Tau is a microtubule-associated protein widely expressed in central and peripheral nervous system. The main function of normal tau protein mainly includes promoting the formation of microtubules, followed by maintaining the stability of microtubules, which all play an important role in maintaining the integrity of neuron cytoskeleton and axoplasmic transport. MAPT is located on autosomal 17q21, which spans 16 exons. In addition, the mRNA of MAPT is able to be selectively cleaved by exons 2, 3, and 10 to produce six different types of protein isomers. Exons 9-12 encode four microtubule binding motifs with 31 or 32 amino acid repeats located at the c-terminal, which are the binding regions of tau protein and microtubules. By selective shearing of exon 10, tau isomers with 3 (exon 10 absent) or 4 (exon 10 visible) repeats are produced, known as 3R-tau or 4R-tau [45, 46].

Abnormal phosphorylation and gene defects of Tau protein are inseparable from the occurrence and development of various degenerative diseases in the nervous system. This kind of sporadic or inherited degenerative diseases in the nervous system is collectively referred to as tau proteopathy. Multiple tau diseases associated with clinical FTD (FTD-Tau) are found and defined now (Pick disease, PSP, CBD, FTDP-17). In addition, 39 tau gene mutations were found: exon 1 (R5H, R5L), exon 9 (K257T, L266V, G272V and 1260V), Exon 10 (P30IL, P301S, S305N, S305S, delK280, delN296, N279K, L284L, N296H, N296N, G303V), 5′ binding site of exon 10 (+3, +11–14, +16, +19), exon 11 (L315R, S320F, K317W), Exon 12 (G335V, G335S, G335R, V337M, E342V, S352L, and K369I), and exon 13 (G389 R, R406W, T427M) [47–50].

A familial correlation between FTD accompanied with Parkinson's disease and chromosome 17q21-22 was found in a patient. Subsequently, several autosomal dominant FTD families linked to 17q21-22 with similar clinical, neuropathological, and genetic characteristics were found, and it was named FTDP-17. Multiple missense and deletion mutations of tau gene coding region and intron have been found in chromosome 17 of FTDP-17 patients, which will lead to changes in tau function, excessive phosphorylation, aggregation of insoluble Tau in brain tissue, destruction of microtubule system, degeneration of nerve cells, deletion and eventually frontotemporal dementia, and Parkinson's syndrome. However, a small number of familial FTD-tau positive cases did not show MAPT mutations. Some FTD patients have been identified as being associated with mutations in the presenin-1 gene (PSEN1). Dermaut et al. reported a 52-year-old early-onset dementia patient who was completely consistent with the clinical diagnosis of FTD, which was caused by PSEN1 G183V mutation. Autopsy neuropathology showed severe frontotemporal lobe atrophy, positive tau staining, presence of Pick cells, and Pick bodies, but no *β* amyloid deposition, which was consistent with the pathology of Pick disease. Another article reported that one FTD family was associated with PSEN1 M146L mutation, which showed both Pick disease and AD neuropathological changes [1, 45]. At present, the mechanism of PSEN1 gene mutation in some FTD subtypes is not clear.

### 3.4. VCP Gene

Inclusion body myositis and hereditary FTD in Paget bone disease share a correlation with 9 pI3.2-pI2, and the pathogenic gene is VCP. VCP is a member belonging to the AAA-ATPase superfamily; with its function of molecular chaperones, this protein also involves in the progression of ubiquitin-dependent endoplasmic-reticulum-associated protein degradation (ER AD), stress response, programmed cell death, cell membrane Fusion, nuclear envelope reconstruction, and the reassembly of Golgi bodies after mitosis [6]. However, the cause of neurodegeneration and TDP-43 accumulation caused by VCP mutations remains unclear, possibly due to changes in ubiquitin-dependent protein degradation. Twelve missense mutations of VCP have been found in 29 families. In the study of FTD cases with Paget bone disease, it was found that the cerebral cortex of lesions contains a large number of Nils and DNs, accompanied by a small amount of NCIs, which are consistent with the pathological characteristics of HLD -TDP4 type [51].

### 3.5. CHMP2B Gene

Mutation in CHMP2B genes is associated with FTD. In recent years, a Danish FTD family was identified to be associated with CHMP2B gene, and its authenticity was further confirmed in a Belgian FTD family. CHMP2B protein was a component of Escort 3 complex, and it was involved in the transport of cytoplasmic denaturated or damaged proteins to Golgi body degradation process. Studies have found that the mutation site of CHMP2B is located in exon 6, where one of the bases G is converted into C, but the specific pathogenic mechanism is still unclear. CHMP2B protein is a component of Escort 3 complex involved in the transport of cytoplasmic denaturated or damaged proteins to Golgi body degradation process [52, 53].

## 4. Outlook

In recent years, great achievements have been made in the research on the neuropathology and pathogenic genes of FTD, but many problems remain, such as chromosome 9 linked familial FTD-tau; furthermore, more pathogenic genes might be discovered. Currently, functions of GRN and TDP-43 in the nervous system need further elucidating. Research on FTD treatment is expected to increase in the future, especially in search of better diagnostic markers and treatment responses. These studies will also pave the way for larger omics studies, despite the preliminary evidence of abnormalities in metabolomics and lipidomics in FTD so far is relatively scarce and focused on only proteomics. With the in-depth researches on the neuropathology and molecular genetics of FTD pushed forward, there would be new methods provided for clinical diagnosis and treatment of FTD.

## Data Availability

The data that support the study are all in the article.
